# Relating Standardized Automated Perimetry Performed With Stimulus Sizes III and V in Eyes With Field Loss Due to Glaucoma and NAION

**DOI:** 10.1167/tvst.13.12.8

**Published:** 2024-12-05

**Authors:** David Szanto, Michael Wall, Luke X. Chong, Mark J. Kupersmith

**Affiliations:** 1 Department of Neurology, Icahn School of Medicine at Mount Sinai, New York, NY, USA; 2 Department of Ophthalmology and Visual Sciences, University of Iowa, Iowa City, IA, USA; 3 School of Medicine, Deakin University, Geelong, Australia; 4 Departments of Neurology, Ophthalmology and Neurosurgery, CNIIC, Icahn School of Medicine at Mount Sinai, New York, NY, USA

**Keywords:** visual field, stimulus size 3, stimulus size 5, censoring

## Abstract

**Purpose:**

Standard automated perimetry (SAP) visual field (VF) results are more repeatable using Goldmann stimulus size V (size V) in eyes with moderate/severe deficits due to glaucoma. There are few reports relating VFs using stimulus size V and III, typically used in the clinic for glaucoma, and none for non-arteritic anterior ischemic optic neuropathy (NAION). We hypothesized that we could compare and relate the VFs with both stimuli for glaucoma and NAION.

**Methods:**

We utilized 1992 same-day pairs of size III and size V SAP VFs using the 24-2 strategy for eyes with glaucoma or NAION. We explored the optimal threshold to censor the raw sensitivities, prior to calculating age-standardized total deviations (TDs). We determined the mean and standard deviation of the differences among all TD pairs. We computed a line of best fit to determine closeness to the line of unity.

**Results:**

The ideal censoring conversion threshold was 21 decibel (dB) for size III and 24 dB for size V. The difference between size V and size III censored (0.0 ± 1.9 dB) and uncensored (0.4 ± 2.6 dB) TD pairings highly correlate with each other (*r*^2^ = 0.70, *P* < 0.001). The line of best fit from these pairings has a slope of 0.92, which is close to that of the line of unity (m = 1).

**Conclusions:**

Censoring plus age correction is a valid method of comparison between size III and size V SAP VFs with moderate to severe VF loss due to optic nerve disorders.

**Translational Relevance:**

Size III and size V TDs are comparable in clinical practice.

## Introduction

Standard Automated Perimetry (SAP) is the most common method used for visual field (VF) testing to measure visual sensitivities at multiple points to detect deficits in the central 30 degrees of vision. The Goldmann stimulus size III (size III) is widely used but subject to useful dynamic range limitation in eyes that have moderate to severe deficits.^1^ The stimulus size V (size V), which uses a larger stimulus, has a wider useful dynamic range and is more reliable for testing eyes with more advanced glaucoma VF loss and also has better test-retest repeatability.^2^

A key challenge is comparing or converting the VF results obtained with different stimulus sizes, particularly for longitudinal data or inter-patient comparisons. Converting VFs obtained with size V to one that would have been obtained with size III can standardize data, making it easier to track disease progression and compare results across studies and clinical settings. However, this conversion process has not been standardized due to the differences in sensitivity and response characteristics between the two stimulus sizes.

Censoring sensitivities below a certain threshold is necessary because data points below about 20 decibel (dB) levels are dominated by noise, making them unreliable and unrepeatable.^3^ Within this range, there is high retest variability which distorts statistical measures and obscures meaningful patterns. Censoring involves adjusting all sensitivity values below a specific threshold to that threshold value. Although the exact cutoff for the useful dynamic range is debated (somewhere below between 17 and 25 dB), setting these low sensitivity values in both stimuli to a predefined threshold should minimize variability with more consistent and interpretable data.^4^^–^^7^

Glaucoma and non-arteritic anterior ischemic optic neuropathy (NAION) are both leading causes of vision impairment in adults.^8^^,^^9^ Both cause irreversible vision loss ranging from mild VF defects to blindness, and can significantly impact the quality of life; they are monitored using VF testing.^8^^,^^9^ Because many patients with both diseases have severely depressed VFs, size V could be used to better monitor the disease progression. This would be particularly helpful in clinical trials that investigate therapies for eyes with moderate to severe VF deficits. Recently, new methods to analyze patterns of VF loss have complemented more global measures of VF loss. Quantifying the specific losses in regions of interest should lead to more precise monitoring and gauging the effects of therapy. Relating these patterns for size V and size III in the same patient or participant is needed. However, we currently cannot directly compare size V VFs to size III VFs, particularly as size III VFs are more likely used earlier in the disease course for glaucoma when it is mild.

This study explored whether we could determine a reasonable threshold censoring level for each point in the 24-2 VF and conversion factor of size V to size III VFs in individuals with moderate to severe VF loss due to glaucoma and NAION. We used two existing datasets that contained both size III and size V data for the same individuals.

## Methods

This study was approved by the Institutional Review Board of the Icahn School of Medicine at Mount Sinai and required no additional consent as the data used were de-identified and derived from participants who had consented for use of their data at multiple study institutions. The study was conducted according to the tenets of the Declaration of Helsinki.

## Study Design and Participants

### Glaucoma

Glaucoma size III and size V data were received from a trial investigating differences in variability between differently sized perimetric stimuli and their ability to discriminate between healthy and damaged VFs in patients with glaucoma. The study compared abnormal test locations across different stimuli sizes to compare findings and extend the analysis to the size modulation perimetry, and was performed at one site in Iowa City, Iowa. This study involved data (previously reported) from 120 participants with glaucoma with moderate to severe VF loss who underwent same-day VF testing using both 24-2 SITA-Standard size III and full threshold size V at the University of Iowa Department of Ophthalmology and Visual Sciences.^10^ Participants were included if they had glaucomatous optic disc changes with abnormal conventional automated perimetry and were diagnosed with primary, secondary, or normal-tension glaucoma with no other vision-affecting diseases. Exclusion criteria included cataract causing visual acuity (VA) worse than 20/30, pupil size less than 2.5 mm, age under 19 years, or being pregnant at the time of study entry.

### Non-Arteritic Anterior Ischemic Optic Neuropathy

NAION data were received from the Quark207 trial, a multinational, prospective, 5-armed randomized controlled trial beginning in 2015 conducted across multiple sites in Australia, China, Germany, India, Israel, Italy, Singapore, and the United States. Its aim was to investigate the safety and efficacy of a biologic in individuals, ages 50 to 80 years, diagnosed with NAION within 14 days of vision loss, meeting study entry criteria.^11^ Inclusion criteria involved best-corrected VA (BCVA) in the study eye with ≥15 Early Treatment Diabetic Retinopathy Study (ETDRS)-letter score at presentation examination.^11^ The study included 729 participants, who were using the Humphrey Field Analyzer with the 24-2 SITA-Standard size III and full threshold size V, which was added after recruitment began. The two stimulus types were tested on the same day. Raw sensitivity values in decibels were recorded for each test. VFs were measured at screening, day 1 of enrollment, 2 months, 6 months, and up to 1 year. There were same-day VFs using both stimuli for participants at month 6 (493), month 12 (414), and various unscheduled times (32).

#### Data Censoring and Conversion

We determined censoring thresholds by comparing the average difference of censored TD values between the results for both stimuli and selecting the thresholds for each stimulus that minimized this difference. We then censored sensitivities by replacing values below the defined threshold with that value. We then converted the sensitivities to age-corrected total deviation (TD) values using a normative database for both stimuli. We also determined the optimal censoring thresholds for each disease separately to demonstrate that the data can be effectively combined in a threshold analysis, defined as the pair of censoring thresholds that results in the average censored TD difference being closest to zero.

#### Data Visualization and Analysis

We performed all statistical analyses in a Jupyter notebook with Python version 3.8.8. All visualizations were done with the open-source python module “matplotlib.”^12^ We plotted pairs of TDs for all points in all VFs (103,584 pairings) as well as the line of unity (y = x) showing the perfect agreement of the points. We also plotted the line of best fit and computed a coefficient of determination. We calculated mean and standard deviations between pairs of TDs, focusing on both uncensored and censored pairs (where at least one pairing is censored) by condition. We performed a paired *t*-test on the difference of all TD pairings.

## Results

We investigated a total of 1992 VFs from 706 participants where 120 had moderate to severe glaucoma and 586 had NAION. In participants with glaucoma, the mean age was 67.8 ± 9.3 years and 39% were men, and, for participants with NAION, the mean age was 61.3 ± 7.7 years and 69% were men (see the Table). The mean TD after censoring in participants with glaucoma for size III and size V was −4.7 ± 3.7 dB and −4.2 ± 3.9 dB, respectively, and for NAION it was −7.2 ± 3.4 dB and −7.3 ± 3.8 dB, respectively. The median BCVA for NAION was 70 letters, with the first quartile at 53 letters and the third quartile at 83 letters with the range extending from 55 to 90 letters. The median BCVA for glaucoma was 80 letters, with the first quartile at 80 letters and the third quartile at 85 letters. The range extended from 55 to 90 letters.

**Table.** Scatterplot of Uncensored TD Pairs Between Size III and Size V

	# VF Pairs	Age, Y	% Male	% Right Eye
Glaucoma (*n* = 120)	**1053**	**67.8 ± 9.3**	**39%**	**53%**
**NAION (*n* = 586)**	**939**	**61.3 ± 7.7**	**69.3%**	**51%**

Demographic information for participants with glaucoma and non-arteritic anterior ischemic optic neuropathy.

**Figure 1.**
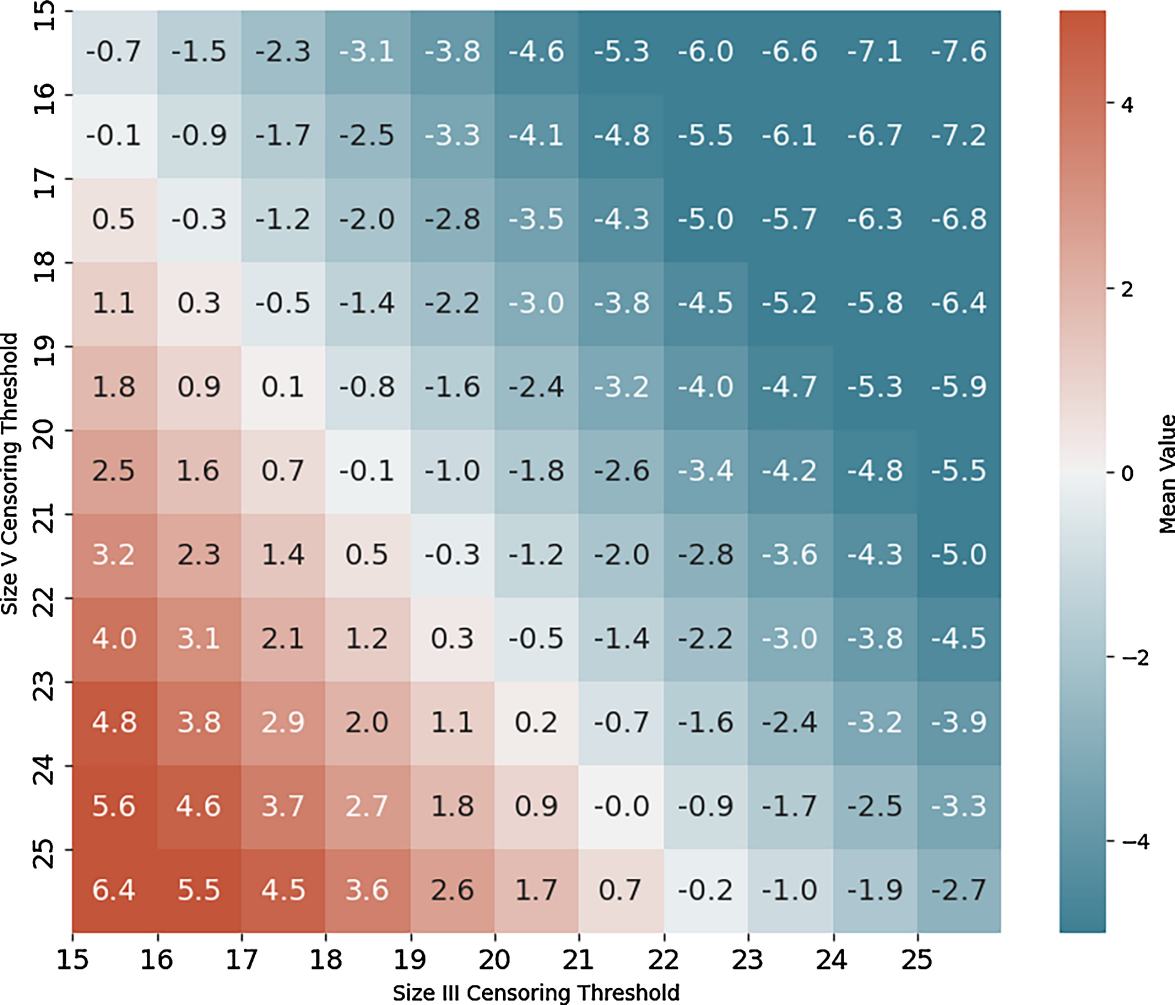
**Mean TD differences between size III and size V pairs across censoring thresholds.** Heatmap depicting the average total deviation difference between pairs of stimulus size III and stimulus size V points across varying censoring thresholds where at least one stimulus was censored. The optimal censoring thresholds were identified at 21 dB for size III and 24 dB for size V.

We discovered that the optimal censoring threshold was 21 for size III and 24 for size V, which provided the lowest mean difference and low standard deviation (Fig. 1, Supplementary Fig. S1).

**Figure 2.**
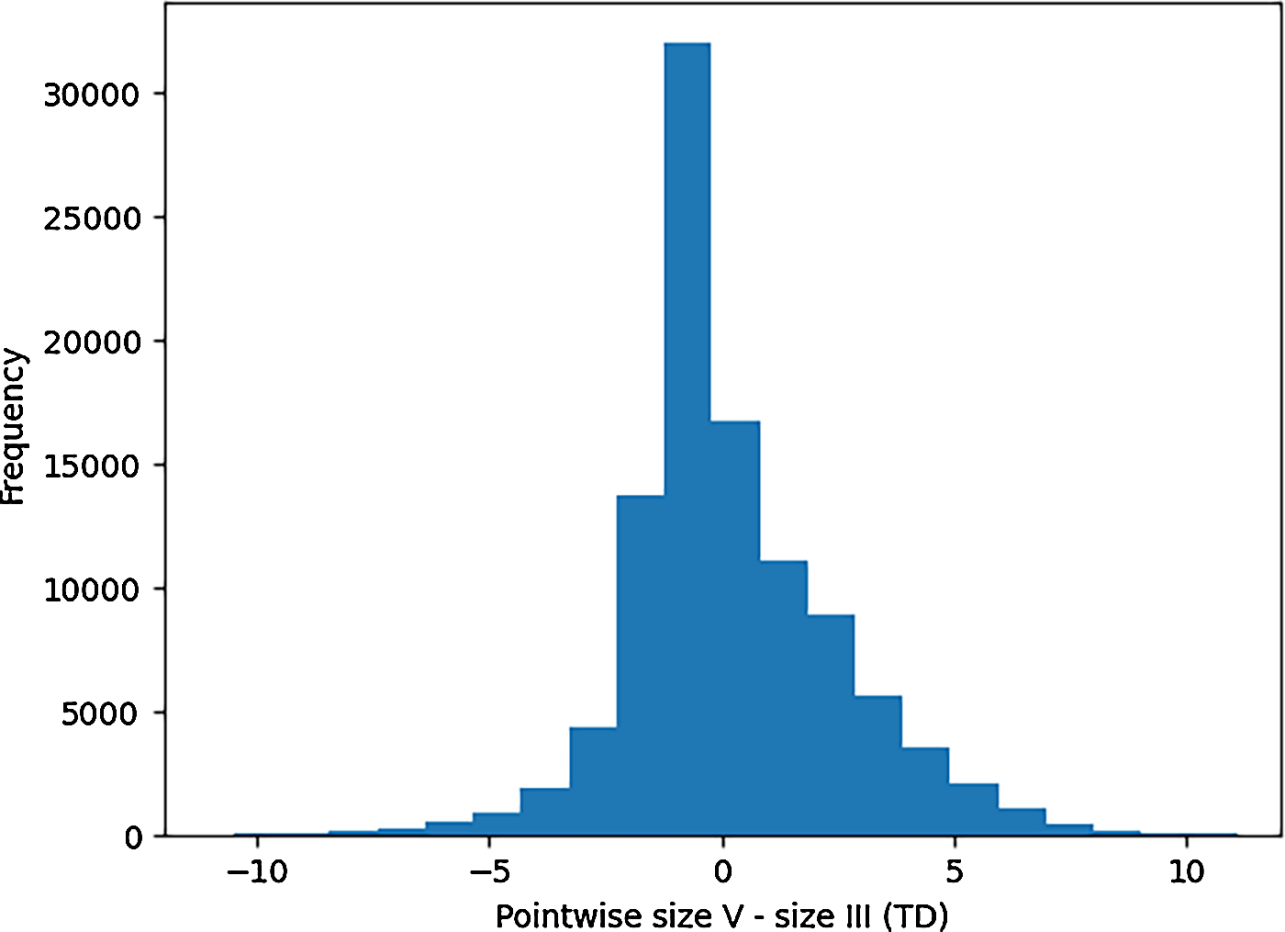
**Pointwise TD differences between size V and size III.** Histogram illustrating the distribution of pointwise total deviation differences between stimulus size V and stimulus size III visual fields. The histogram is centered around 0, indicating comparable differences between the two stimuli across the tested points.

**Figure 3.**
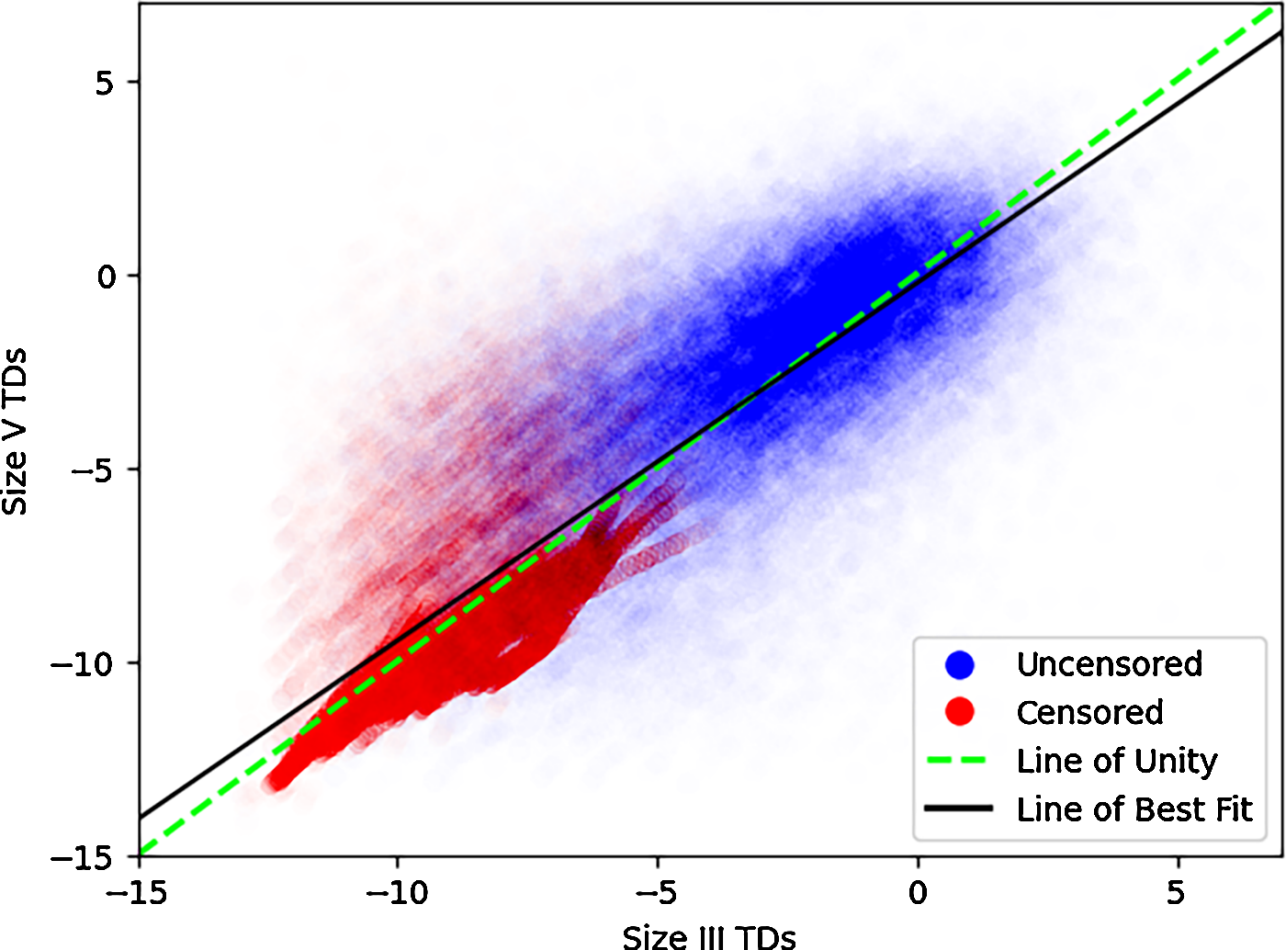
**Scatterplot of TD pairs between size III and size V.** Pairs of total deviation values from stimulus size III and stimulus size V visual fields for non-arteritic anterior ischemic optic neuropathy and glaucoma. Data points are colored based on whether at least one of the pair is censored. The *dotted green line* represents the line of unity (y = x). The *black line* of best fit (y = −0.24 + 0.92 x) demonstrates a substantial linear relationship between the two stimuli (*r*^2^ = 0.70). Data points are made transparent for improved visibility and to illustrate relative density.

**Figure 4.**
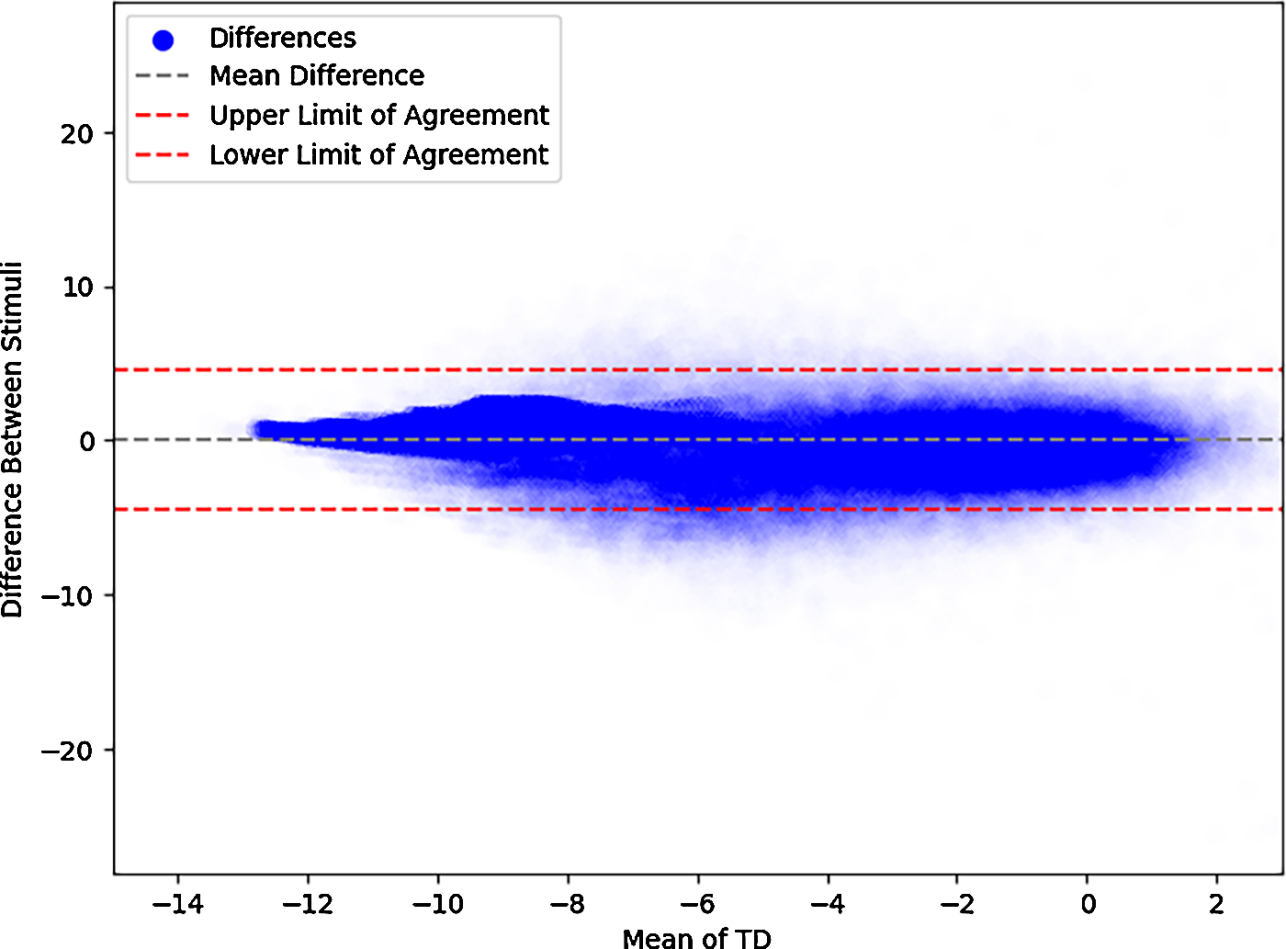
**Bland-Altman plot comparing visual field measurements between stimuli.** Bland-Altman plot depicting the agreement between stimuli sizes III and V based on total deviation values. The x-axis represents the mean of the values for each pair of measurements, whereas the y-axis shows the difference between the two stimuli sizes. The *solid blue points* indicate the individual differences. The *dashed gray line* represents the mean difference centered near 0, whereas the *dashed red lines* indicate the upper and lower limits of agreement of two standard deviations from the mean. The plot demonstrates consistent variability across most of the range, with decreased variability seen at lower mean TD values, due to data censoring.

**Figure 5.**
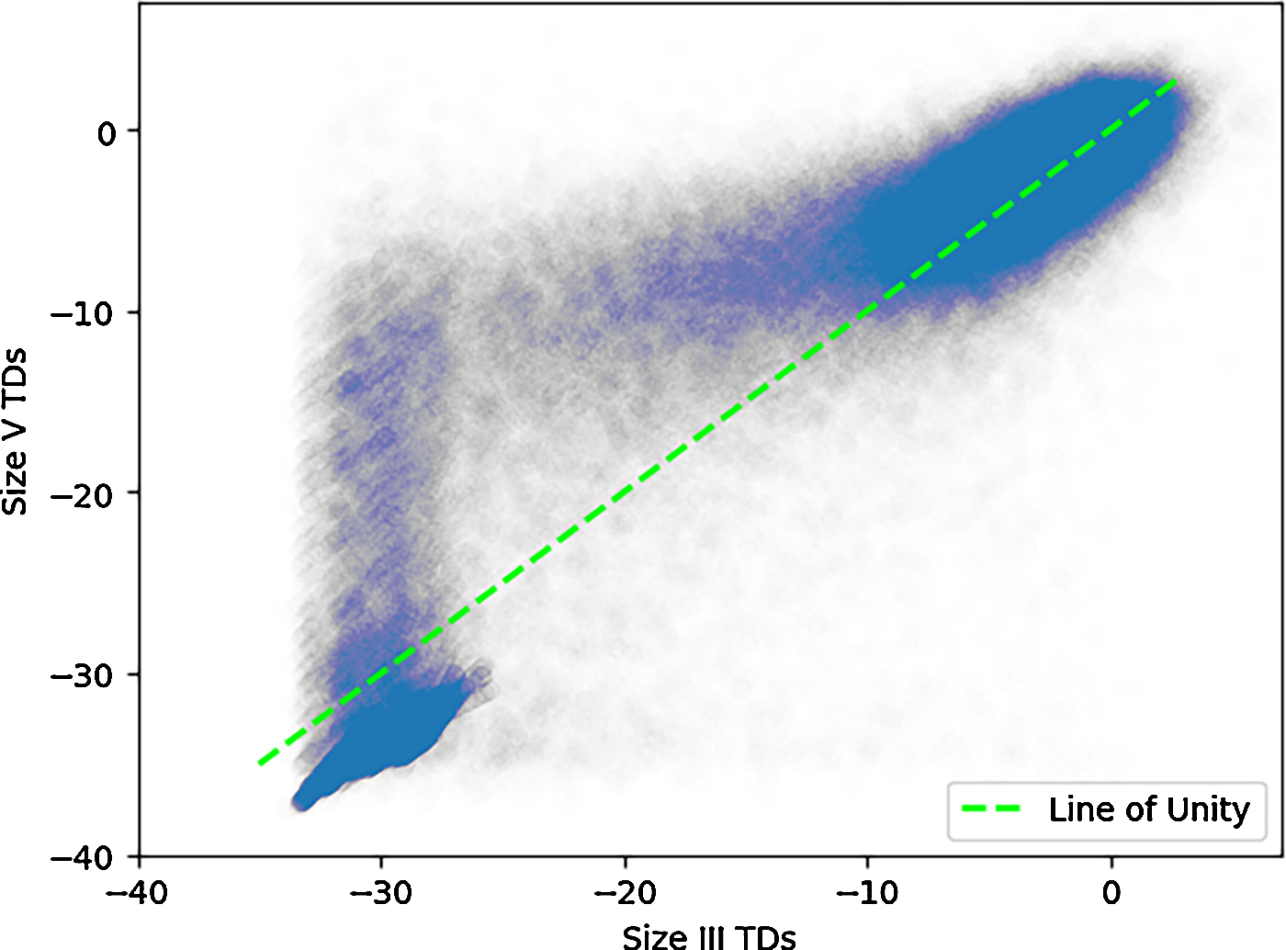
**Scatterplot of uncensored TD pairs between size III and size V.** Pairs of uncensored total deviation values from stimulus size III and stimulus size V visual fields for non-arteritic anterior ischemic optic neuropathy and glaucoma. The dotted green line represents the line of unity (y = x). Beyond a certain threshold, points increasingly deviate from the line of unity, highlighting point variability in uncensored measurements. The axes range from −40 dB to 5 dB. Data points are made transparent for improved visibility and to illustrate their relative density. SD of differences between size III and size V pairs across censoring thresholds.

Overall, the average difference between size V and size III TD pairings for uncensored pairs was 0.4 ± 2.6 dB, and a censored pair difference of 0.0 ± 1.9 dB (Fig. 2). A paired *t*-test analyzing differences of TDs show a statistically significant nonzero (0.1 dB) difference between the two stimuli (*P* < 0.001). Glaucoma VF differences between size III and size V were remarkably consistent over all subtypes, showing an uncensored pair difference of 0.4 ± 2.6 dB and a censored pair difference of 0.0 ± 1.9 dB. NAION VFs consist of an uncensored pair difference of 0.2 ± 2.9 dB and a censored pair difference of −0.3 ± 1.8 dB. Notably, 63% of pairings in NAION were censored. We plotted a line of best fit along all pairs of points, and we found that it was similar to the line of unity (Fig. 3). There was a high correlation between TD pairings (*r*^2^ = 0.70). A Bland-Altman plot shows that the agreement between the two stimuli is generally stable, with no changes in variability in all but the most negative TD values (Fig. 4). Opting to not censor TD pairings reveals that, beyond a certain threshold, data points increasingly deviate from the line of unity in an unpredictable way (Fig. 5).

In performing threshold analysis on just glaucoma, we found that the optimal censoring threshold was 20 dB for size III and 22 dB for size V. Using these thresholds for glaucoma TD pairings, the resultant line of best fit had a slope of 0.91 with high correlation (*r*^2^ = 0.68). The same analysis on only NAION TD pairings resulted in censoring thresholds of 19 dB for size III and 22 dB for size V, and the resultant line of best fit had a slope of 0.91 with a high correlation (*r*^2^ = 0.67).

## Discussion

We established that censoring and converting sensitivities to TDs enables direct comparison between size III and size V. The average difference for censored and uncensored data is marginal, but the retest variability is markedly reduced using censoring. Determining an optimal censoring threshold for VFs for each stimulus improves the comparison between stimuli particularly in VFs with regions of major sensitivity loss.

We determined that the optimal censoring thresholds for size III and size V are 21 dB and 24 dB for this data set of 2 different optic nerve disorders. These thresholds provided the lowest mean differences and low standard deviations, and are both within a reasonable range, allowing for better comparison between eyes with severely depressed VFs. Our combined threshold pair falls within 2 dB to those calculated for each disease separately, and the lines of best fit for the combined and single disease computations have close values for all slopes (0.91, 0.91, and 0.92) and *r*² values (0.68, 0.67, and 0.70) for glaucoma, NAION, and combined, respectively.

A paired *t*-test revealed a small, but significant, average difference between TD pairings. However, this number is very close to zero, and considering same-day testing variability, this number has minimal clinical relevance. Prior studies have shown that the inter-test variability for specific points range from 1.3 dB to 3.0 dB for size III and 1.2 dB to 2.0 dB for size V.^7^ Thus, the two stimuli should be comparable in clinical practice if censoring and age corrected TDs are used. This allows clinicians to switch from a test using one stimulus size to another without clinically meaningful discrepancies affecting clinical decisions. For NAION and glaucoma, the average TD difference and standard deviation were very similar (as well as between censored and uncensored pairs), suggesting that our methodology may broadly apply for a variety of optic nerve disorders.

The line of best fit for TD pairings closely followed the line of unity. Coupled with its high *r*^2^ value, suggests there is high agreement between size III and size V across different visual field severities. The variability in our uncensored data scatterplot highlights the necessity of censoring to maintain reliability and consistency.

The difference in thresholds observed when analyzing optimal thresholds for NAION and glaucoma individually compared to when combined stems from the fact that the optimal thresholds for each condition are not optimal for the other. The threshold determination for glaucoma of 20 (size III) and 22 (size V) results in an approximate censored TD difference of +0.1 dB, whereas for NAION, the same thresholds lead to an average TD difference of −0.9 dB. When combined, the weighted average TD difference is −0.5 dB. Whereas thresholds of 21 (size III) and 24 (size V) are not optimal for either condition individually, they are deemed sufficiently effective when analyzed in combination. This highlights that although individual thresholds may be optimal for one condition, they may not equally benefit the other, leading to a less effective combined threshold. However, it is important to note that these differences are clinically insignificant and underscore the necessity of censoring, then selecting thresholds that allow for accurate and direct comparisons across both conditions.

This study has clinical implications. Clinicians can choose between size III and size V based on the degree of visual field damage without compromising diagnostic accuracy. Our optimal censoring thresholds also improve the comparability of VFs not only within the same patient but across different patients as well in the setting of severe vision loss, aiding in better disease management.

Our study had limitations typical of retrospective analyses. First, widespread population data sets for VFs performed using stimulus V are lacking and are not included in the current Humphrey perimeters. Differences in testing protocols or equipment settings between the two datasets may have introduced variability that would affect the comparability of size III and size V results, particularly due to the presence of multiple testing sites in both groups. Of course, having NAION VFs performed at 80 sites supports the potential for real-world applicability of our method. We also only tested VFs of participants with NAION and glaucoma with substantial damage so we had relatively fewer data points near a TD of 0 and above. We also treated each VF observation as independent to perform linear regression, although this may limit the generalizability and interpretation of these results.

Our study shows that usage of normative databases for size III and size V, in conjunction with censoring, allows for the direct comparison between VFs using either of the two stimuli. Future directions will include validation studies for diverse patient populations and optic nerve diseases. Comparison of different stimulus sizes may also aid in reliable tracking of disease progression. Leveraging both size III and size V stimuli within the same dataset will also eliminate the need to segregate data by Goldmann stimulus size.

## Supplementary Material

Supplement 1

## References

[1] Wall M, Brito CF, Woodward KR, Doyle CK, Kardon RH, Johnson CA. Total deviation probability plots for stimulus size V perimetry: a comparison with size III stimuli. *Arch Ophthalmol*. 2008; 126(4): 473–479. 18413515 10.1001/archopht.126.4.473

[2] Gardiner SK, Demirel S, Goren D, Mansberger SL, Swanson WH. The effect of stimulus size on the reliable stimulus range of perimetry. *Transl Vis Sci Technol*. 2015; 4(2): 10. 10.1167/tvst.4.2.10 PMC4378323 25883877

[3] Gardiner SK, Swanson WH, Goren D, Mansberger SL, Demirel S. Assessment of the reliability of standard automated perimetry in regions of glaucomatous damage. *Ophthalmology*. 2014; 121(7): 1359–1369. 24629617 10.1016/j.ophtha.2014.01.020 PMC4082764

[4] Wall M, Zamba GK, Artes PH. The effective dynamic ranges for glaucomatous visual field progression with standard automated perimetry and stimulus sizes III and V. *Invest Ophthalmol Vis Sci*. 2018; 59(1): 439–445. 29356822 10.1167/iovs.17-22390 PMC5777662

[5] Gardiner SK, Swanson WH, Demirel S. The effect of limiting the range of perimetric sensitivities on pointwise assessment of visual field progression in glaucoma. *Invest Ophthalmol Vis Sci*. 2016; 57(1): 288–294. 26824408 10.1167/iovs.15-18000 PMC4736987

[6] Pathak M, Demirel S, Gardiner SK. Reducing variability of perimetric global indices from eyes with progressive glaucoma by censoring unreliable sensitivity data. *Transl Vis Sci Technol*. 2017; 6(4): 11. 10.1167/tvst.6.4.11 PMC5518759 28736685

[7] Bittner AK, Mistry A, Nehmad L, Khan R, Dagnelie G. Improvements in test–retest variability of static automated perimetry by censoring results with low sensitivity in retinitis pigmentosa. *Transl Vis Sci Technol*. 2020; 9(12): 26. 10.1167/tvst.9.12.26 PMC7683849 33244446

[8] Kingman S. Glaucoma is second leading cause of blindness globally. *Bull World Health Organ*. 2004; 82: 887–888. 15640929 PMC2623060

[9] Hattenhauer MG, Leavitt JA, Hodge DO, Grill R, Gray DT. Incidence of nonarteritic anterior ischemic optic neuropathy. *Am J Ophthalmol*. 1997; 123(1): 103–107. 9186104 10.1016/s0002-9394(14)70999-7

[10] Wall M, Doyle CK, Zamba KD, Artes P, Johnson CA. The repeatability of mean defect with size III and size V standard automated perimetry. *Invest Ophthalmol Vis Sci*. 2013; 54(2): 1345–1351. 23341012 10.1167/iovs.12-10299

[11] Kupersmith MJ, Fraser CL, Morgenstern R, et al. Ophthalmic and systemic factors of acute nonarteritic anterior ischemic optic neuropathy in the Quark207 Treatment Trial. *Ophthalmology*. 2024; 131(7): 790–802. 38211825 10.1016/j.ophtha.2024.01.011

[12] Hunter JD. Matplotlib: a 2D graphics environment. *Comput Sci Eng.* 2007; 9(3): 90–95.

