# Supplementary material for: Relating Standardized Automated Perimetry Performed With Stimulus Sizes III and V in Eyes With Field Loss Due to Glaucoma and NAION
Source: Transl Vis Sci Technol. 2024 Dec 5;13(12):8. doi: 10.1167/tvst.13.12.8 (PMC11622156; doi:10.1167/tvst.13.12.8)
Supplement: Supplement 1 [file tvst-13-12-8_s001.pdf]

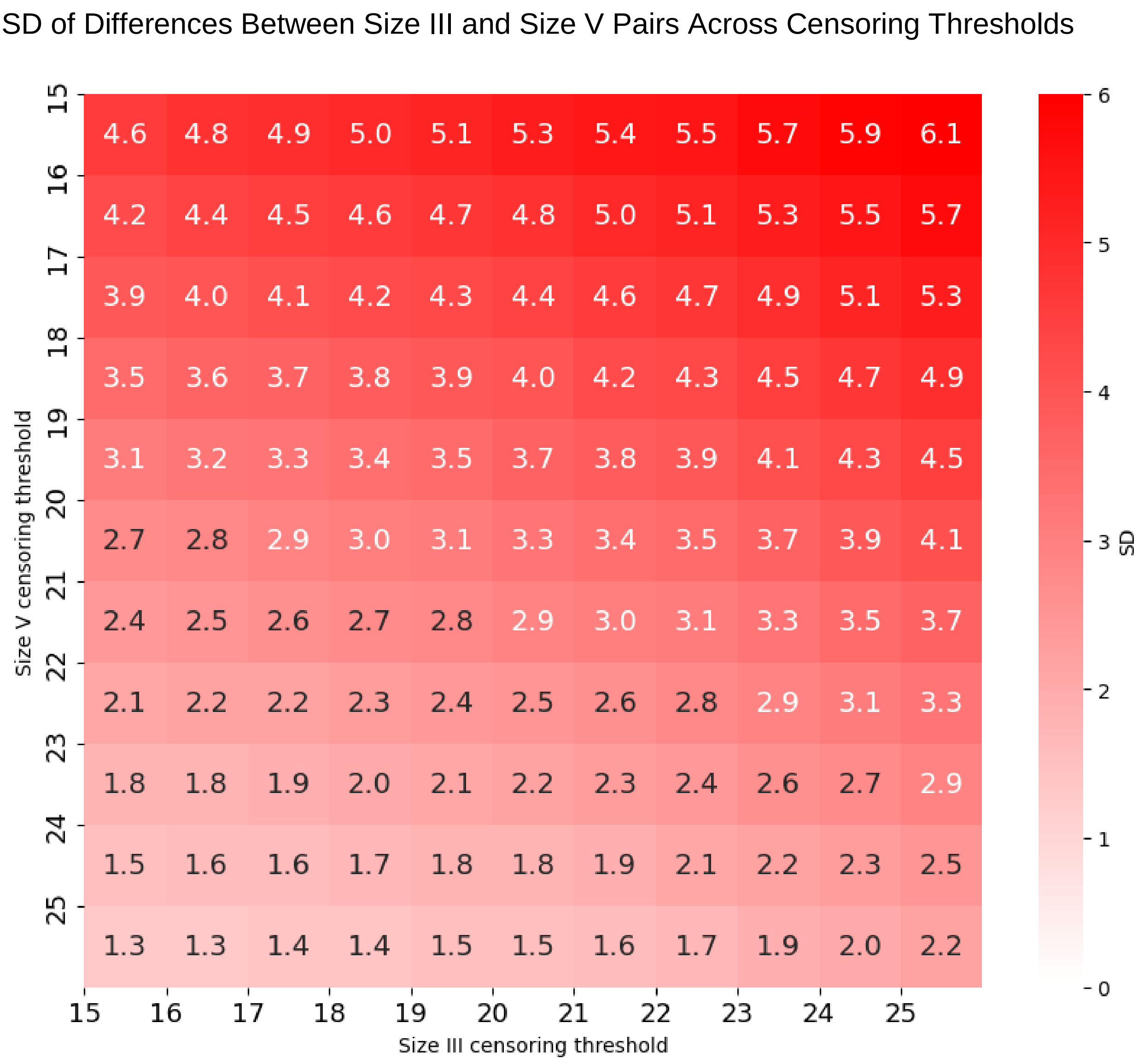

Supplemental Figure 1. Heatmap depicting the standard deviation of total deviation differences between pairs of stimulus size III and stimulus size V points across varying censoring thresholds where at least one stimulus was censored. For the optimal censoring threshold of 21 dB for stimulus size III and 24 for stimulus size V, the standard deviation is 1.9 dB.
